# Porous Silicon Nanocomposites with Combined Hard and Soft Magnetic Properties

**DOI:** 10.1186/s11671-016-1617-0

**Published:** 2016-09-13

**Authors:** Klemens Rumpf, Petra Granitzer, Herwig Michor

**Affiliations:** 1 Institute of Physics, Karl Franzens University Graz, Universitaetsplatz 5, A-8010 Graz, Austria; 2 Institute of Solid State Physics, Vienna University of Technology, Wiedner Hauptstr. 8, Vienna, Austria

**Keywords:** Porous silicon, Electrodeposition, Magnetic nanostructures, Magnetic interactions

## Abstract

Magnetic nanostructures of two ferromagnetic metals have been combined within porous silicon, and the magnetic switching behavior of the resulting porous silicon/metal nanocomposite has been modified by varying the arrangement. The two magnetic materials are Ni and Co, whereas Co is the magnetic harder one. These “hard/soft” magnetic nanocomposites have been achieved by two different routes. On the one hand, double-sided porous silicon has been used whereas one side has been filled with Ni nanostructures and the other one with Co nanostructures. On the other hand, Ni and Co have been deposited within one porous layer alternatingly. The filling of the pores has been carried out by electrodeposition with varying the deposition parameters. In systems which offer two distinct slopes of the hysteresis curves due to the different saturation behavior of the two types of deposited metal, magnetic exchange coupling is not present. For samples which show smooth hysteresis curves exchange, coupling between the Ni and Co nanostructures seems to be present. The aim is to control especially the structure size of the soft and the hard magnetic materials and the distance between them at the nanoscale to optimize exchange coupling resulting in a maximum energy product.

## Background

The utilization of low-dimensional structures becomes more and more important due to the miniaturization of devices and also due to the novel arising nanoscopic properties. The fabrication of such nanostructures is often carried out by nanopatterning using lithographic methods. On the other hand, self-assembling techniques are of great interest due to the uncomplex and low-cost fabrication process. Quite common are nanoparticles grown on a substrate by self-organization. Also, three-dimensional arrays of nanostructures (nanowires, nanotubes) have been formed without prestructuring in using hexagonal arranged porous alumina as matrices [1]. Also, quasi-regular arranged porous silicon has been used for the incorporation of metal deposits [2, 3]. In this context, ferromagnetic nanostructures are an important part in basic research but also in nanotechnological applications such as magneto-optical devices, magnetic sensors, or high-density data storage [4]. A further ambition is the production of exchange-coupled nanostructures usable as permanent magnets. One approach is the self-organization of hard and soft magnetic nanoparticles which are tunable in their size and inter-particle distance [5].

Considering the achieved nanocomposites, magnetization reversal processes with the concomitant domain wall motion within the deposited metal nanostructures, the interactions among them, and also transport phenomena like magnetoresistance in spin valves are of great interest. Magnetic materials in the nanometer scale exhibit changed properties compared to bulk material and therefore offer great potential for novel nanotechnological applications. The nanoscopic systems consist either of particles or wires with magnetic properties dependent on their geometry and arrangement. For technical application of the system, the magnetic nanostructures should be ferromagnetic at room temperature. In some cases, a high anisotropy between the two magnetization directions, perpendicular and parallel to the surface, is of interest and thus needle-like structures are favorable due to their high demagnetizing field. One method to achieve low-dimensional structures is the deposition of metal nanostructures on patterned surfaces or into porous membranes with channels perpendicular to the surface, and therefore, the metal structures exhibit a high density with respect to the sample surface. Templates like porous alumina or polycarbonate foils are usually electrochemically fabricated and afterwards filled with a magnetic material by electrochemical deposition. In commercial microelectronics, most devices are based on silicon technology and thus for process compatibility, a silicon substrate is a good precondition for applicability.

In the present work, a combined porous silicon/“hard-soft” magnetic nanocomposite is investigated and the results concerning the magnetic properties with respect to the switching behavior are figured out.

## Methods

Two kinds of porous silicon templates have been fabricated by anodization of an n^+^ silicon wafer. On the one hand, a silicon wafer has been porosified on one side with an average thickness of the porous layer of 40 μm. On the other hand, ultrathin wafers have been etched on both sides (front and back) in using an electrolytic double tank cell. The thickness of the porous layers was around 20 μm on each side. As anodization electrolyte, an 10 wt% aqueous hydrofluoric acid solution has been used. To achieve pore diameters of about 60 nm, a current density of 100 mA/cm^2^ has been applied. The etching rate was around 4 μm/min.

The prepared porous silicon templates offer a morphology in the mesoporous regime with a quasi-regular pore arrangement. Within the pores of these templates, nanostructures consisting of two different magnetic materials have been incorporated by electrodeposition. The double-sided porous silicon has been filled with Ni on one side and with Co on the other side, also using a double tank cell. Depending on the polarity of the applied current, either Ni on the one side or Co on the other side is deposited. For the Ni deposition, a current density of 25 mA/cm^2^ with 0.05 Hz and for the Co deposition, a current density of 20 mA/cm^2^ with 0.1 Hz has been applied. In the case of the one-sided sample, both metals have been electrochemically deposited alternately inside the pores resulting in a stacked arrangement of Co and Ni nanostructures. As electrolytes, a Ni and Co metal salt solution (NiCl_2_, NiSO_4_, CoSO_4_) has been used. The deposition time for each metal was up to 10 min. Magnetic characterization of the samples has been performed by SQUID (superconducting quantum interference device) magnetometry (field range ± 6 T, temperature range 4–300 K).

## Results and Discussion

**Fig. 1:**
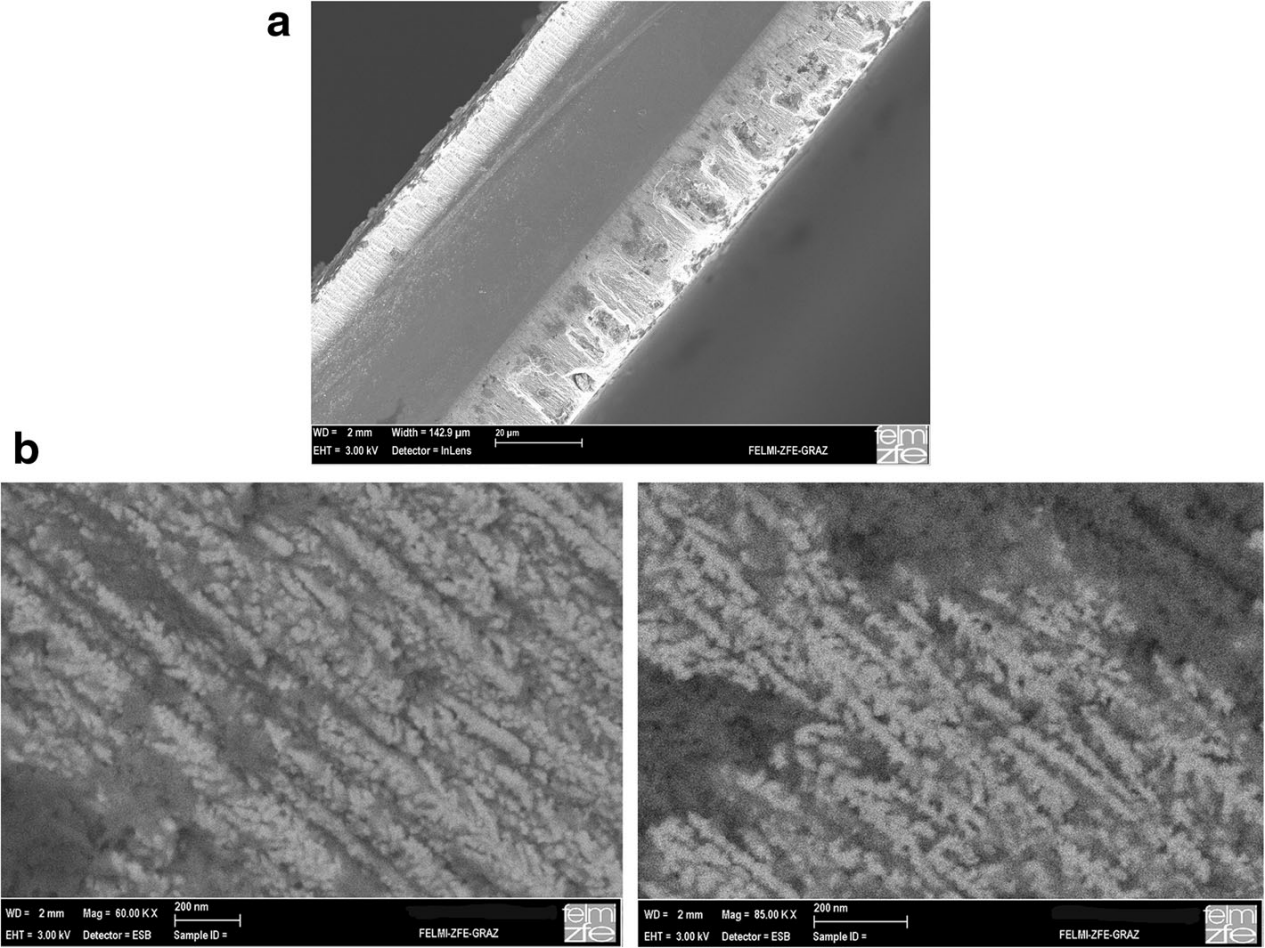
**a** Cross-sectional scanning electron microscopy (SEM) image of a thinned n^+^ wafer showing a porous layer on each side filled with Ni and Co, respectively. **b** SEM images showing Ni deposits within the porosified layer on the one side (*left*) and Co structures deposited within the porous silicon layer of the other side (*right*)

The deposited magnetic nanostructures consist of Co and Ni, whereas Co is the magnetic “harder” material. Considering the magnetization measurements of double-sided porous silicon containing Co nanostructures on one side and Ni nanostructures on the other side, one sees that the magnetic behavior is composed of two terms. The first one is due to the softer magnetic material, Ni, which exhibits a saturation magnetization of 0.6 T (bulk Ni). The second one is due to the harder magnetic material, Co, which exhibits a saturation magnetization of 1.7 T (bulk Co). Thus, the hysteresis curves of the investigated nanocomposite systems show two different slopes corresponding to the two different materials. Figure 1a shows a double-sided porous silicon sample filled with Ni on one side (left) and Co on the other side (right). In Fig. 1b, the corresponding zoomed areas exhibiting the Ni and Co structures within the pores can be seen.

**Fig. 2:**
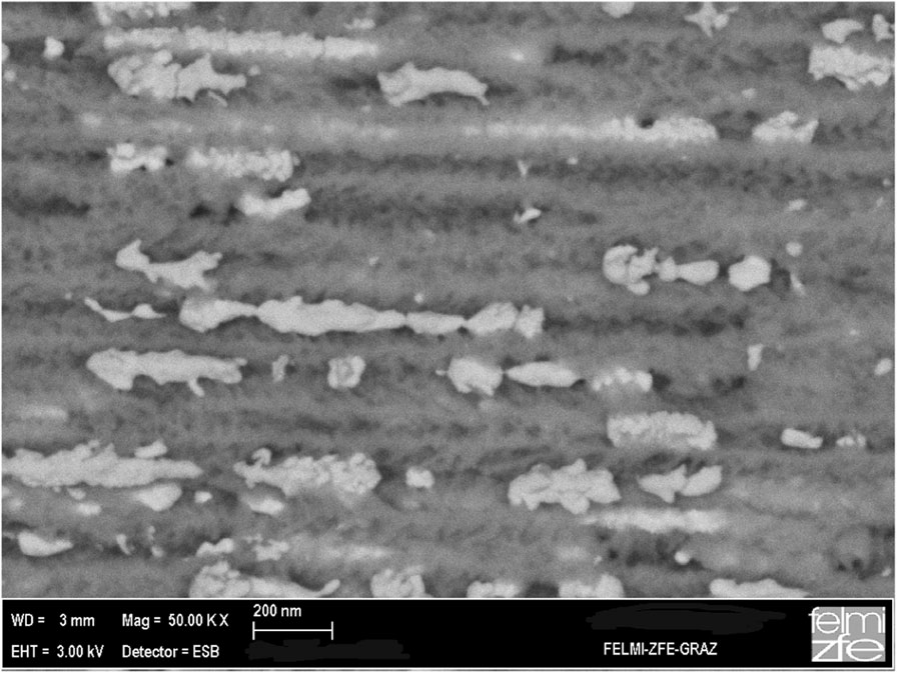
Cross-sectional scanning electron micrograph of a porous silicon sample containing Ni as well as Co nanostructures. Since the atomic weight of Co and Ni is similar, the elements cannot be distinguished by the back-scattered electrons (BSE)

In the case of double-sided samples filled with Ni and Co, magnetic interactions can only occur between nanostructures within one layer. The distance between the two porous layers is in the range of 30 μm which makes dipolar coupling negligible. For non-interacting deposits, the coercivity *H*_C_ of the nanocomposite depends mainly on the size and shape of the deposited metal structures. In the case of a dense spatial distribution with magnetically coupled deposits, additionally, the interaction influences *H*_C_. The magnetic anisotropy is mainly due to shape anisotropy and increases with the elongation of the deposited metal structures. However, the deposition of densely packed structures results in a wire-like behavior caused by magnetic coupling between them. Generally, the coercivity decreases with the elongation of the metal deposits. In Table 1, an example for the variation of the coercivity and squareness (magnetic remanence *M*_R_/saturation magnetization *M*_S_) of Ni deposited within porous silicon is given. Considering double-sided samples containing two metals, the magnetic characteristics are a combination of both metals. Considering single-sided samples consisting of stacked Co/Ni nanostructures, the magnetic behavior depends on the kind of deposition. In the case of using one combined electrolyte bath, consisting of the Ni- and the Co-salt, different voltages have been applied for the two metals. The samples show also a clear kink in the hysteresis curve demonstrating that exchange coupling between the two materials is excluded which can be ascribed to a distance greater than 2 nm between the different nanostructures or an oxide formation separating them. Details of this circumstance are currently under investigation. An example of such a sample with combined filling can be seen in Fig. 2.

**Table 1:** Coercivity and squareness in dependence on the elongation of Ni deposits

Shape and elongation of Ni deposits	*H*_C_[Oe]	*M*_R_/*M*_S_
Spherical, ~60 nm	520	0.57
Ellipsoidal, ~500 nm	350	0.48
Wire-like, >1 μm	260	0.35

**Fig. 3:**
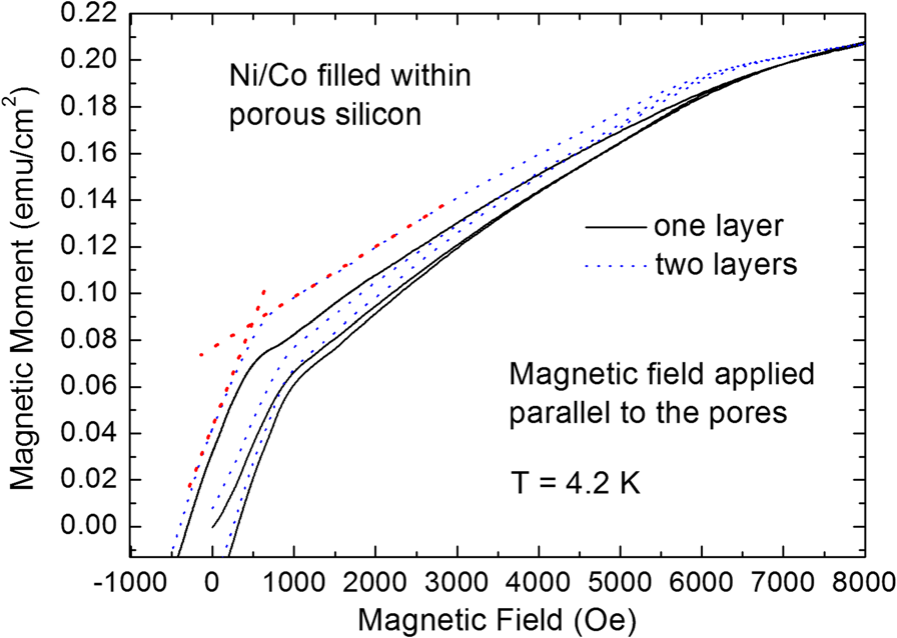
Field-dependent magnetization of two types of samples. *Blue-dotted line* corresponds to the double-sided porous silicon sample with Ni filling on the one and Co filling on the other side. The *black full line* corresponds to the sample with one porous layer filled with both materials out of one electrolyte solution alternatingly

Figure 3 shows the comparison of field-dependent magnetization measurements of a double-sided etched porous silicon sample, filled with Ni on one side and Co on the other one, and a one-sided etched sample with both materials deposited simultaneously. One sees that both samples offer a clear kink corresponding to the different magnetic switching behavior of Ni and Co, whereas the slope is a little steeper in the case of the double-sided sample. This behavior indicates that in the case of filling both materials in one porous layer, a small amount of oxide can be formed. First, up to a field of 500 Oe, the ferromagnetic behavior of Ni is dominant, and above the saturation of the Ni-structures, the behavior of the Co structures, which are saturated at higher fields, becomes more distinctive.

**Fig. 4:**
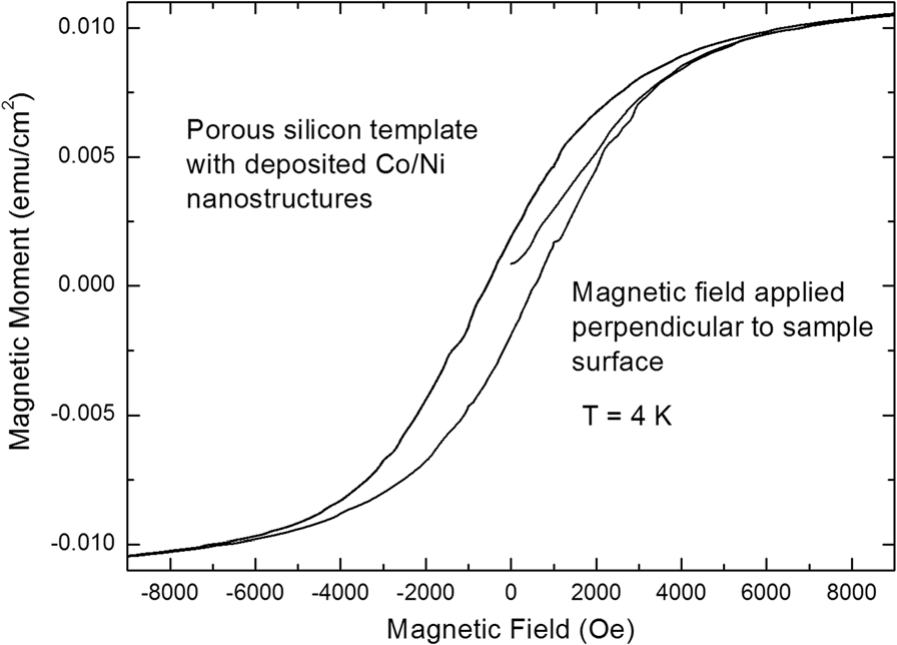
Field-dependent magnetization measurements of porous silicon containing alternatingly deposited Co and Ni nanostructures out of two different solutions

In performing the deposition of the two magnetic materials from two different electrolyte baths (Watts electrolyte and CoSO_4_ solution), field-dependent magnetization shows a smooth hysteresis which indicates the presence of exchange coupling between the Ni and Co deposits (Fig. 4). In tuning the size of the two kinds of deposits, the exchange coupling can be influenced and thus optimized to achieve samples with a maximum energy product which could give rise to an array of permanent nanomagnets.

## Conclusions

The presented “hard/soft” magnetic nanocomposite is fabricated during a low-cost two-step electrochemical process whereas double-sided as well as one-sided porous silicon acts as template. Considering the magnetic behavior of double-sided systems and single-sided systems gained from a combined electrolyte solution, two characteristic terms are observed. The first one is caused by the magnetic properties of the “softer” magnetic metal (Ni) and the second one is caused by the higher saturation magnetization of the deposited “harder” magnetic nanostructures (Co). In the case of depositing Co and Ni alternatingly from a Watts-solution and CoSO_4_-solution, a smooth hysteresis curve is observed due to exchange coupling between the different metal deposits. Furthermore, the magnetic properties are determined by the geometry of the deposits and their arrangement and thus the coercivity, remanence, and magnetic anisotropy of the samples (both types) can be modified by varying the size and shape of the Ni and Co deposits. Therefore, the magnetic characteristics of the specimens can be broadly tailored, as desired. The magnetic properties of the resulting nanocomposites are not only correlated with the size, shape, and spatial distribution of the deposited metal structures but also strongly depend on the filling ratio between the “softer” (Ni) and “harder” (Co) magnetic materials as well as on the way how the electrodeposition is performed (from a combined or separate solutions).

## Abbreviations

BSE: Back scattered electrons
*H*_C_: Coercivity
*M*_R_: Magnetic remanence
*M*_S_: Saturation magnetization
SEM: Scanning electron microscopy
SQUID: Superconducting quantum interference device
